# Rethinking risk: The prognostic power of coronary plaque in young women

**DOI:** 10.1016/j.ajpc.2026.101649

**Published:** 2026-04-22

**Authors:** Bede N. Nriagu, Todd C. Villines, Garima Sharma

**Affiliations:** a Inova Schar Heart and Vascular, Inova Health System, Falls Church, VA, 22031, USA; b Division of Cardiovascular Medicine, Department of Medicine, University of Virginia School of Medicine, Charlottesville, VA, 22908, USA

The contemporary understanding of many diseases has been shaped by clinical trials conducted predominantly in men, resulting in disease models derived largely from male-based data [1]. In cardiovascular disease (CVD), this imbalance has created persistent gaps in our understanding of sex-specific coronary artery disease (CAD) patterns and outcomes.

Recognition of coronary atherosclerotic plaque has emerged as a critical component of cardiovascular risk assessment. Contemporary imaging studies using coronary computed tomography angiography (CCTA) have demonstrated that the presence, burden, and composition of coronary plaque provide powerful prognostic of cardiovascular risk [2]. Importantly, women demonstrate fundamentally distinct atherosclerotic phenotypes compared to men, with less necrotic core and calcium, less plaque rupture, smaller lumens and similar plaque burden [1]. The biological mechanisms for these differences likely reflect complex interactions between sex hormones, vascular biology, and atherosclerotic cell signaling. For example, in postmenopausal women, the decline in estrogen levels is associated with an increased risk of CVD [3]. Similarly, sex hormone binding globulin, free testosterone, and estrone have each been linked to differences in carotid plaque burden, echogenicity, and markers of plaque stability [3]. Taken together, loss of estrogen accelerates endothelial dysfunction, inflammation, and atherosclerosis development.

In this issue of the Journal, Asif et al. present a large retrospective single-center analysis of 3813 young (<60 years; mean age 49 years and 36% women), predominately low-risk patients who underwent clinically indicated CCTA from 2007–2019 [4]. A substantial proportion - 35% of men and 19% of women - were asymptomatic. CAD severity was quantified using validated measures including segment involvement score (SIS), Duke Prognostic Index (DPI), coronary artery calcium (CAC), and number of vessels with non-obstructive (<50%) or obstructive (>50%) disease. Outcomes included all-cause mortality and a secondary composite of acute coronary syndrome (ACS) and nonfatal myocardial infarction over a median 7.4-year follow-up.

As expected, overall event rates were low, and men - despite being slightly younger, had more extensive plaque burden, consistent with prior literature [5]. The most compelling finding, however, is the relative difference in risk associated with plaque among women. Women experienced greater mortality risk per incremental increase in plaque burden across SIS, DPI, and CAC. Discriminatory performance of these measures was consistently superior in women, with multivariable C-statistics of 0.729–0.752 versus 0.680–0.690 in men. This paradox reinforces an emerging principle: the presence of coronary plaque in women represents a more ominous biological signal than equivalent disease in men [6]. Interpretation of CAD severity, therefore, must incorporate sex-specific patterns of disease and risk.

Recent data support this concept. In the CONFIRM2 registry, every 50-mm³ increase in AI-quantified plaque volume conferred a 17.7% increase in major adverse cardiovascular events in women (median age 61 years) as compared with only 5.3% in men [6]. The current study extends these observations to a younger cohort (mean age 51years), a critical window that corresponds to the perimenopausal transition when plaque acceleration begins. A contemporary Chinese cohort of 16,300 patients undergoing CCTA revealed a roughly 20-year delay in atherosclerosis onset in women, yet a nonlinear, rapidly accelerating trajectory thereafter - distinct from the linear progression observed in men [5]. Plaque accumulation in women showed minimal increase until age 45, rose modestly from 45–50, then tripled between 50–55 years, nearly coinciding with the average age of natural menopause [5]. Once plaque is present in postmenopausal women, it carries disproportionate prognostic weight compared with men [6].

A particularly concerning secondary finding of the study was the lower use of lipid-lowering therapy in women both before and after CCTA. This treatment gap mirrors extensive evidence showing that women are less likely to receive guideline-directed preventive therapies, even after myocardial infarction [7]. In the present cohort, men were more frequently initiated on therapy after CCTA, whereas women, with less extensive but more prognostically adverse plaque, were treated less aggressively. This mismatch between clinical decision-making and sex-specific risk may partially explain the observed mortality differences and highlights the limitations of current risk assessment tools, which frequently underestimate risk in younger women.

The clinical implications are clear. First, CCTA findings in women should be interpreted with sex-specific thresholds. The presence of any plaque in a younger or perimenopausal woman should prompt heightened concern and aggressive risk factor modification. Second, comprehensive risk assessment in women must extend beyond traditional calculators and incorporate female-specific risk factors such as polycystic ovary syndrome (PCOS) and pregnancy-associated conditions, including age of menarche and menopause, parity, infertility, use of assisted reproductive technology (ART), spontaneous pregnancy loss, and adverse pregnancy outcomes (APOs), that increase future risk of CVD[7]. Third, CAC and CCTA may be particularly useful in intermediate-risk women or those with risk-enhancing factors, as supported by the Coronary Artery Calcium Score: Use to Guide Management of Hereditary Coronary Artery Disease (CAUGHT-CAD) trial demonstrating that CAC-guided therapy reduces atherogenic lipid levels and slows plaque progression [8].

The authors appropriately acknowledge key limitations, including the single-center design, low adverse event numbers (particularly among women), and lack of measured high-risk plaque features, plaque volume quantification, menopausal status, and post-CCTA treatment changes. The absence of menopausal status is especially important, given its central role in plaque biology and timing. Future studies should integrate detailed hormonal profiling and female-specific risk factors to elucidate mechanisms underlying sex-based differences in atherosclerosis progression and prognosis. Further study is needed to better quantify the prognostic implications of high-risk plaque features in women which, while less common than in men, may represent more risk when present in women relative to men. In a study by Lee et al. of 1255 subjects who underwent serial CCTA at ≥2-year interval, the prevalence of high-risk plaques was greater in men than women (31% vs. 20%; p < 0.001) with women less likely to develop new high-risk plaque on subsequent CCTA imaging [9]. Fortunately, recent studies utilizing advanced coronary plaque volume quantification have attempted to define reference ranges, much like the important work published using CAC, specific to patients of differing age and sex [2]. However, further work is needed to expand the populations studied to ensure broad representation of society, such that reference ranges are not skewed or biased against younger patients with smaller volumes of plaque that convey important long-term risk.

Nonetheless, this study contributes important evidence supporting the growing recognition that cardiovascular risk in women requires a fundamentally different approach. In an era of expanding use of CT-based imaging, the finding that plaque conveys disproportionately greater risk in women underscores the need for sex-specific prevention strategies. A uniform, sex-neutral approach to CAD risk assessment risks overlooking younger women who stand to benefit most from early intervention. Recognizing and responding to these sex differences is essential to closing persistent gaps in cardiovascular outcomes.

**Figure:**
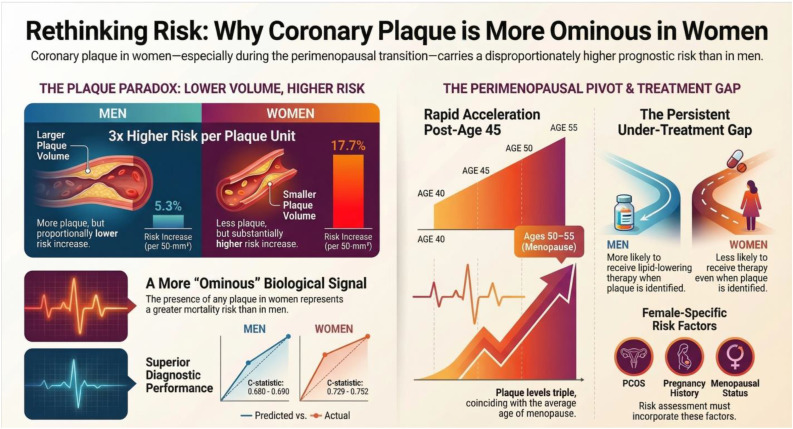
**Central Illustration:** Rethinking Risk: The Prognostic Power of Coronary Plaque in Young Women.

## CRediT authorship contribution statement

**Bede N. Nriagu:** Writing – review & editing, Writing – original draft, Visualization. **Todd C. Villines:** Writing – review & editing, Supervision. **Garima Sharma:** Writing – review & editing, Supervision.

## Declaration of competing interest

The authors declare that they have no known competing financial interests or personal relationships that could have appeared to influence the work reported in this paper.
